# Triglyceride/High-Density Lipoprotein Cholesterol Ratio Is Associated with In-Hospital Mortality in Acute Type B Aortic Dissection

**DOI:** 10.1155/2020/5419846

**Published:** 2020-04-08

**Authors:** Yang Zhou, Guifang Yang, Huaping He, Xiaogao Pan, Wen Peng, Xiangping Chai

**Affiliations:** Department of Emergency Medicine, The Second Xiangya Hospital of Central South University, 139 Renmin road, Changsha, Hunan Province 410011, China

## Abstract

**Background:**

Triglyceride/high-density lipoprotein cholesterol (TG/HDL-c) ratio varies with vascular and other metabolic diseases. However, its role in acute type B aortic dissection is not well understood. In the current study, we evaluated the relationship between TG/HDL-c ratio and in-hospital mortality in type B aortic dissection.

**Methods:**

We performed a retrospective analysis of consecutive patients between January 2015 and December 2018, by targeting dependent (TG/HDL-c ratio) and independent (in-hospital mortality) variables. TG/HDL-c ratio was determined as a division of TG levels by HDL-c levels.

**Results:**

Of 523 patients in the study, we found a mean age of 55.00 ± 11.74 years, 15.68% of them being female. A fully-adjusted model revealed a positive relationship between TG/HDL-c ratio and in-hospital mortality in acute type B aortic dissection after adjusting confounders (OR = 2.08, 95% CI 1.32 to 3.27). This relationship was also nonlinear, with a point of 2.05. OR values (and confidence intervals) for the right (>2.05) and left (≤2.05) sides of the inflection point were 1.0 (0.580-1.26, *P* = 0.983) and 3.17 (1.54-6.57, *P* = 0.001), respectively.

**Conclusions:**

The TG/HDL-c ratio and in-hospital mortality in type B AAD have a nonlinear relationship among Chinese population. This ratio increased in-hospital mortality when it is less than 2.05.

## 1. Introduction

Acute aortic dissection (AAD) is a rare, life-threatening condition associated with high morbidity and mortality rates [1, 2]. In-hospital mortality rates of patients with AAD is up to 27.4% [2, 3], with type B AAD reported to account for one-third of all AAD cases [4]. Despite the recent improvements in management of type B AAD, mortality rates and postoperative complications of this disease remain high [5]. Studies have reported that medical treatment has been accepted as standard management for type B AAD, although its 5-year total survival rate stands at 60% [6]. It is, therefore, urgent to investigate the risk factors of type B AAD mortality and develop an effective intervention. Studies have reported several risk factors for type B AAD in-hospital mortality, including age, hypertension, ischemic complications, and genetic factors [7].

To date, dyslipidemia is a well-documented risk factor for stroke [8, 9], metabolic syndrome [10], and cardiovascular disease [11, 12]. Moreover, results from a meta-analysis suggested that lipid-modifying therapy is a protective factor on mortality after abdominal aortic aneurysm repair [13]. A cross-sectional study implicated dyslipidemia aortic stiffness [14], with international guidelines stating that lipid profiles should be managed [15]. Atherogenic dyslipidemia, a combination of low high-density lipoprotein cholesterol (HDL-c) and high triglyceride (TG) with elevated small dense low-density lipoprotein (LDL) particles and apolipoprotein B, is an important part of the metabolic syndrome and a powerful predictor of cardiovascular disease [16–18]. However, the costs of performing the LDL phenotyping are expensive and have not been standardized [19, 20]. As a possible alternative, the TG/HDL-c ratio has been considered an easily available atherosclerotic marker [21]. Previous studies have reported that a higher TG/HDL-C ratio is related with LDL phenotype B, small insulin resistance, HDL particles, and cerebro/cardiovascular diseases [16]. In the current study, we aimed to evaluate the relationship between TG/HDL-c ratio and in-hospital mortality in patients with type B AAD.

## 2. Methods

### 2.1. Participants

**Figure 1:**
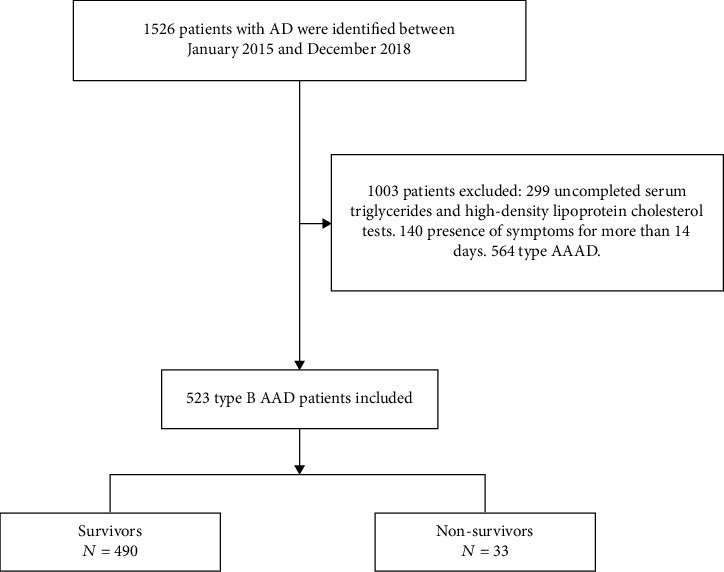
Flow chart of patient enrollment.

This was a retrospective study, in which we nonselectively and consecutively evaluated 1526 patients between January 2015 and December 2018 at the Second Xiangya Hospital of Central South University. The Diagnostic process was mainly based on guidelines proposed by ESC 2014 on the treatment and diagnosis of aortic diseases [22]. Among the patients, 140 of them who showed presence of symptoms for more than 14 days were excluded. Similarly, those whose covariate data was missing (uncompleted serum triglyceride or high-density lipoprotein cholesterol tests) were also excluded. According to the Stanford classification, which is based on anatomical categorization [23], a total of 564 type A AAD patients were excluded. Finally, 523 type B AAD patients were enrolled in the study (Figure 1). The study was approved by the Institutional Review Board at the Second Xiangya Hospital of Central South University.

### 2.2. Clinical Assessment

We evaluated risk factors and demographic characteristic, including age, gender, body mass index (BMI), hypertension, stroke, smoking, diabetes milieus (DM), atherosclerosis, Marfan syndrome (MFS), chronic renal insufficiency (CRI), systolic blood pressure (SBP), diastolic blood pressure (DBP), operation, and mortality. Laboratory examinations including white blood cells count (WBC), hemoglobin (Hb), high-density lipoprotein cholesterol (HDL-c), platelet count (PLT), serum triglyceride (TG), alanine transaminase (ALT), uric acid (UA), creatinine (Cr), aspartate aminotransferase (AST), and troponin T (TnT) were analyzed in blood collection from AAD patients in the morning, following 12 hours of fasting. A TG/HDL-c ratio was calculated after by dividing TG levels by HDL cholesterol levels.

### 2.3. Statistical Analysis

Continuous variables were expressed as mean ± standard deviations of the mean (normal distribution) or median with interquartile range (IQR) (Skewed distribution), while categorical variables were expressed as frequencies or percentages. Kruskal-Wallis H (skewed distribution) and One-Way ANOVA tests (normal distribution) or *χ*^2^ (categorical variables) were performed, applied, and compared with Q1, Q2, Q3, and Q4 (TG/HDL-c ratio categorized as quartiles). To investigate the relationship between TG/HDL-c ratios and in-hospital mortality, we performed statistical analyses based on 3 points. First, we use univariate and multivariate linear regression model analyses. A total of three models were established: model I, in which only sociodemographic data were adjusted; model II, the model I added other covariates shown in Table 1. Secondly, in order to solve the nonlinearity of TG/HDL-c ratio and in-hospital mortality, a smooth curve fitting (penalized spline method) was conducted. If nonlinearity was detected, we first calculated the inflection point using the recursive algorithm and then constructed a two-piecewise linear regression on both sides of the inflection point. This was followed by determination of the best fit model on the basis of *P* values for log-likelihood ratio test. The third step involved subgroup analysis based on stratified linear regression models. Continuous variables were initially transformed into categorical variables using the clinical quartile of cut point, followed by interaction tests. Tests for effect modification across subgroup indicators were followed by analysis of likelihood ratios. Sensitivity analyses were then performed, in which the TG/HDL-c ratio was converted to categorical variables for calculation of the *P* for trend to ensure the robustness of the analysis. The aim of this step was to validate the results of TG/HDL-c ratio as continuous variables and to detect nonlinearity. These analyses were carried out in Empower Stats (http://www.empowerstats.com, X&Y Solutions Inc., Boston, MA) [24] and R (http://www.r-projec.org, The R Foundation). Statistical significance was determined at *P* < 0.05.

## 3. Results

### 3.1. Baseline Characteristics and Univariate Analyses

A total of 523 type B AAD patients were enrolled in the study, and this resulted a median age of 55.00 ± 11.74 years. 84.32% of these were male. A flow chart outlining their selection is presented in Figure 1, while baseline features of the enrolled patients are outlined in Table 1. In-hospital mortality was found in 33 (6.31%) patients. Univariate analyses indicated that the outcome variable was associated with CRI, operation, and the levels of Hb, AST, ALT, TG, TG/HDL-c ratio, Cr, UA, and TnT (Table 2).

### 3.2. TG/HDL-c Ratio Increased the In-Hospital Mortality

**Figure 2:**
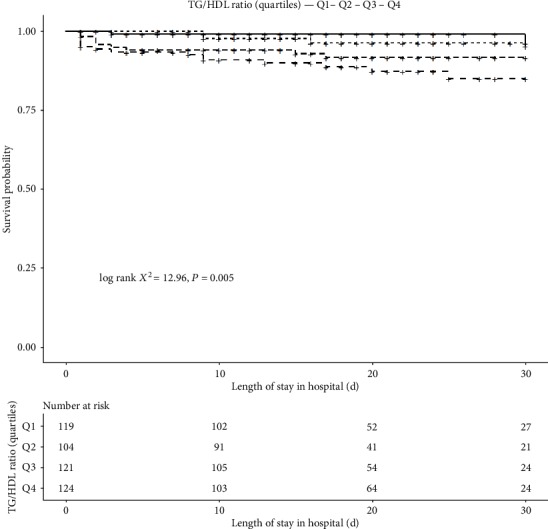
Kaplan-Meier curves for in-hospital survival according to TG/HDL-c (quartiles) in all type B AAD patients.

Results from the Kaplan-Meier analysis showed a significantly higher cumulative in-hospital survival rate in the Q1 relative to the other groups (log-rank *χ*^2^ = 12.96, *P* = 0.005) (Figure 2).

### 3.3. The Relationship between TG/HDL-C Ratio and In-Hospital Mortality

We established three models in order to examine the independent effects of TG/HDL-c ratio on in-hospital mortality after adjusting for confounding factors (Table 3). Model III resulted in a TG/HDL-c ratio (OR = 2.08, 95% CI = 1.32 to 3.27, *P* = 0.002) remained an important predictor of in-hospital mortality after all adjusted covariates are showed in Table 1. We also converted TG/HDL-c ratio from continuous to categorical variable (quartiles), the *P* for the trend of categorized TG/HDL-c ratio in the fully adjusted model matched with the result when TG/HDL-c ratio is a continuous variable. However, when the TG/HDL-c ratio enters the fully-adjusted model as a categorical variable, the trend of the effective value in the different TG/HDL-c ratio group had nonequidistant changes. There may be a nonlinear relationship between TG/HDL-c ratio and in-hospital mortality according to this kind of nonequidistant changes in effect size.

### 3.4. The Nonlinearity of TG/HDL-c Ratio and In-Hospital Mortality

**Figure 3:**
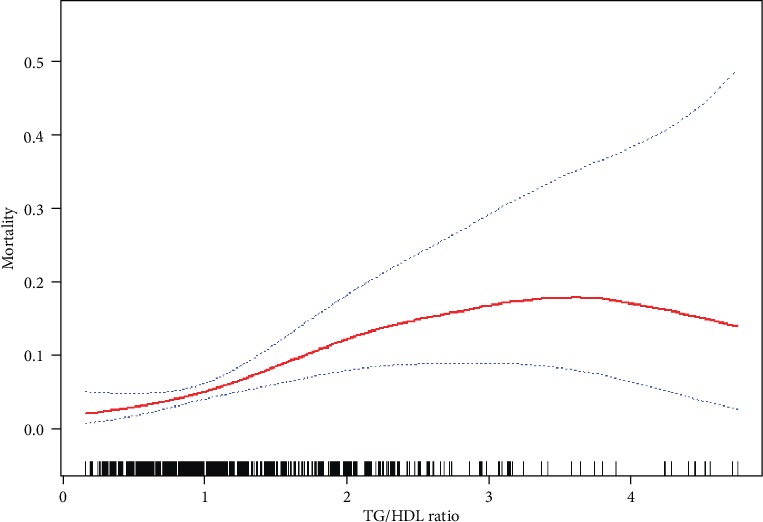
Association between TG/HDL-c ratio and in-hospital mortality. A nonlinear association between TG/HDL-c ratio and in-hospital mortality was found. The solid red line represents the smooth curve fit between variables. Blue bands represent the 95% confidence interval from the fit. All adjusted for gender, age, BMI, hypertension, smoking, atherosclerosis, WBC, PLT, Hb, ALT, AST, Cr, UA, and TnT.

We performed further analysis on the nonlinear relationship of TG/HDL-c ratio with in-hospital mortality (Table 4, Figure 3). Results of the smooth curve indicated nonlinear relationship (adjusted for other covariates listed in Table 1) between TG/HDL-c ratio and in-hospital mortality. We used the two-piecewise linear regression and the linear regression models to fit the association between TG/HDL-c ratio and in-hospital mortality, respectively. The *P* value for the log-likelihood ratio test was 0.017, indicating that the two-side linear regression was more appropriate for fitting the association between TG/HDL-c ratio and in-hospital mortality because it can accurately represent the relationship between them. The inflection point, determined by the two-piecewise recursive algorithm and linear regression, was 2.05. On the left side of the inflection point (TG/HDL − c ratio ≤ 2.05), the effect size and 95% CI were 3.17, 1.54 to 6.57, respectively. On the right side of the inflection point (TG/HDL − c ratio > 2.05), the relationship cannot be observed (1.003 (95% CI 0.8-1.26), *P* = 0.983). Though they are all consistent with an increased risk of in-hospital mortality in the right side of the inflection point, there is a lack of a statistically significant association with the TG/HDL-c ratio.

### 3.5. Subgroup Analysis

In the subgroup analysis, we used the stratification variables, including gender, BMI, DM, age, smoking, hypertension, atherosclerosis, CRI, stroke, WBC, PLT, Hb, ALT, AST, Cr, UA, UA, and operation, to observe the trend of effect sizes (Table 5).

## 4. Discussion

The current study resulted in three main findings. First, we found a positive relationship between TG/HDL-c ratio and in-hospital mortality, after adjusting other covariates. This indicated that an increase in TG/HDL-c ratio produces 2.08-fold increase in in-hospital mortality. Secondly, our analysis revealed nonlinearity between in-hospital mortality and TG/HDL-c ratio. Given that this correlation displayed a ratio-response pattern, our findings may provide clues for the potential pathophysiologic mechanisms. Thirdly, our results confirmed an association between in-hospital mortality and TG/HDL-C ratio in type B AAD in Chinese patients which can be used to screen high-risk individuals. To better understand the trend in this association in different populations, we performed subgroup analysis and found stable results.

Several studies have suggested high levels of TG/HDL-c ratio in cardiovascular diseases across Europe and Asian Pacific populations [25–27]. TG/HDL-c ratio indicates harmful small/dense LDL particles and has been found to have a positive association with atherosclerosis [28]. Moreover, a previous study [14] analyzing lipid parameters of 603 participants to evaluate aortic stiffness found a significant association between aortic stiffness with total cholesterol/HDL, total cholesterol, and non-HDL. Therefore, the cholesterol deposition in the aortic tissues may explain the development of type B AAD or abdominal aortic aneurysm. In additional, there is a large number of evidence that has suggested the role of TG/HDL-c ratio in prediction of poor outcomes in cardiovascular, especially myocardial infarction and ischemic stroke [28, 29]. Furthermore, previous studies have also deduced that the TG/HDL-c ratio has a positive association with the occurrence of ischemic [30], which is potentially the source of high TG/HDL-c ratio in type B AAD patients [31].

Although operation is still the most effective way to avoid in-hospital deaths in patients with AAD, other risk factors for the prediction of in-hospital mortality need to be considered. Our results suggested that TG/HDL-c ratio is one of the risk factors for in-hospital mortality. Clinical therapies should therefore arouse high attention to the ratio with these patients. Increased TG/HDL-c ratios are not only related to aortic stiffness, but also an indicator of inflammatory factors and oxidative stress, which show a risk of type B AAD and a poor prognosis. Recent evidence suggests that inflammatory factors and oxidative stress play a crucial role in the pathogenesis and progression of AAD [32, 33]. For instance, Daiber et al. [34] reported the quality of high-density lipoprotein changes under oxidative stress conditions. Similarly, Kaye et al. [35] found that if the levels of HDL are insufficient, cholesterol and lipids in arterial walls trigger an exacerbated inflammatory response. Meta-analysis reported that low serum lipids may reduce patient mortality after abdominal aortic aneurysm repair [13]. Overall, increased TG/HDL-c ratio in type B AAD is associated with aortic stiffness, inflammatory factors, and oxidative stress.

The advantages of our research are strict statistical adjustment, subgroup analysis, and large research population. However, there are some weaknesses in the study. Firstly, the participants herein were mainly Chinese AAD patients, recruited in central south China. As a result of this sampling, our study may not be generalizable to AAD populations in other regions as factors influencing cholesterol levels may vary. Secondly, we excluded chronic AD and type A AAD patients from analysis; therefore, the study conclusion does not apply to these people. Consequently, because it is a retrospective study, some variables such as physical activity, diet, and daily habits were not achieved. We also were not able to know if the patients had fibrates. Nevertheless, our study results indicate that the TG/HDL-C ratio is a risk factor. If a patient takes lipid-lowering drugs, high TG/HDL-C ratio will have a strong positive association with in-hospital mortality.

## 5. Conclusions

The TG/HDL-c ratio and in-hospital mortality in type B AAD have a nonlinear relationship among Chinese population. This ratio increased in-hospital mortality when it is less than 2.05.

## Figures and Tables

**Table 1:** Baseline characteristics of the cohort (*N* = 523).

Characteristics	Total	TG/HDL-c ratio (mmol/L) (quartiles)				*P* value
		Q1 (<0.71)	Q2 (0.71-1.12)	Q3 (1.13-1.87)	Q4 (>1.89)	
*n*	523	131	130	131	131	
Age (years)	55.00 ± 11.74	57.71 ± 10.46	55.95 ± 12.05	52.54 ± 10.96	53.80 ± 12.81	<0.001
Female	82 (15.68%)	29 (22.14%)	25 (19.23%)	17 (12.98%)	11 (8.40%)	0.010
BMI (kg/cm^2^)	25.47 ± 3.87	24.65 ± 3.10	24.63 ± 4.25	26.91 ± 4.74	25.72 ± 2.80	0.291
SBP	150.73 ± 27.17	155.54 ± 27.35	149.07 ± 26.46	149.57 ± 26.10	148.67 ± 28.47	0.134
DBP	86.02 ± 16.18	87.56 ± 15.81	84.29 ± 14.85	87.06 ± 17.53	85.16 ± 16.37	0.312
Smoking	209 (39.96%)	42 (32.06%)	46 (35.38%)	53 (40.46%)	68 (51.91%)	0.006
DM	25 (4.78%)	9 (6.87%)	2 (1.54%)	11 (8.40%)	3 (2.29%)	0.020
Hypertension	386 (73.80%)	85 (64.89%)	90 (69.23%)	99 (75.57%)	112 (85.50%)	<0.001
Stroke	16 (3.06%)	8 (6.11%)	2 (1.54%)	4 (3.05%)	2 (1.53%)	0.104
Atherosclerosis	51 (9.75%)	11 (8.40%)	10 (7.69%)	12 (9.16%)	18 (13.74%)	0.345
MFS	5 (0.96%)	1 (0.77%)	1 (0.77%)	2 (1.53%)	1 (0.77%)	0.529
CRI	16 (3.06%)	1 (0.76%)	4 (3.08%)	7 (5.34%)	4 (3.05%)	0.201
WBC (×109/L)	10.92 ± 3.76	10.89 ± 3.23	11.41 ± 4.33	10.52 ± 3.49	10.88 ± 3.88	0.401
PLT (×109/L)	195.77 ± 78.14	165.81 ± 55.25	190.14 ± 61.16	210.46 ± 89.90	216.62 ± 89.90	<0.001
Hb (g/L)	125.76 ± 20.60	126.84 ± 17.68	125.72 ± 21.36	125.63 ± 21.86	124.82 ± 21.43	0.923
ALT (u/L)	19.95 (13.50-33.38)	16.60 (11.45-24.55)	18.50 (13.03-28.70)	23.30 (14.33-34.92)	25.80 (16.10-51.30)	<0.001
AST (u/L)	19.50 (15.15-28.60)	20.10 (15.75-26.15)	18.20 (14.85-25.00)	18.85 (14.85-26.28)	22.45 (15.30-37.80)	0.121
Cr (umol/L)	79.10 (65.45-103.88)	72.30 (59.90-88.20)	74.70 (63.30-102.20)	76.20 (63.40-104.05)	92.50 (73.50-122.20)	<0.001
UA (mmol/L)	317.49 ± 111.70	300.27 ± 105.43	332.41 ± 118.87	310.98 ± 103.98	326.31 ± 116.19	0.031
TnT (pg/mL)	7.83 (4.50-14.02)	7.17 (4.59-9.68)	7.46 (4.83-11.98)	6.99 (3.66-13.57)	9.87 (5.53-21.50)	0.001
TG (mmol/L)	1.48 ± 0.85	0.71 ± 0.19	1.06 ± 0.22	1.53 ± 0.43	2.63 ± 0.72	<0.001
HDL-c (mmol/L)	1.19 ± 0.32	1.46 ± 0.33	1.20 ± 0.22	1.06 ± 0.26	1.03 ± 0.26	<0.001
Operation						0.003
No	117 (22.37%)	37 (28.24%)	14 (10.77%)	32 (24.43%)	34 (25.95%)	
Yes	406 (77.63%)	94 (71.76%)	116 (89.23%)	99 (75.57%)	97 (74.05%)	
Mortality						<0.001
Survivor	490 (93.69%)	129 (98.47%)	126 (96.92%)	121 (92.37%)	114 (87.02%)	
Nonsurvivor	33 (6.31%)	2 (1.53%)	4 (3.08%)	10 (7.63%)	17 (12.98%)	

Abbreviations: BMI: body mass index; SBP: systolic blood pressure; DBP: diastole blood pressure; WBC: white blood cells count; PLT: platelet count; Hb: hemoglobin; ALT: alanine transaminase; AST: aspartate aminotransferase; Cr: creatinine; UA: uric acid; TnT: troponin T; DM: diabetes mellitus; MFS: Marfan syndrome; CRI: chronic renal insufficiency; TG: triglyceride; HDL-c: high-density lipoprotein cholesterol.

**Table 2:** Univariate analysis for in-hospital mortality.

Characteristics	Statistics	OR (95% CI)	*P* value
Age (years)	55.00 ± 11.74	1.01 (0.99, 1.04)	0.366
Female	82 (15.68%)	0.33 (0.08, 1.41)	0.135
BMI	25.47 ± 3.87	1.00 (0.89, 1.15)	0.673
SBP	150.73 ± 27.17	0.99 (0.98, 1.01)	0.352
DBP	86.02 ± 16.18	0.99 (0.97, 1.01)	0.479
Smoking	209 (39.96%)	1.45 (0.71, 2.94)	0.304
Diabetes	25 (4.78%)	0.61 (0.08, 4.63)	0.629
Hypertension	386 (73.80%)	1.34 (0.57, 3.16)	0.502
Stroke	16 (3.06%)	1.45 (0.71, 2.94)	0.304
Atherosclerosis	51 (9.75%)	0.92(0.04, 2.06)	0.895
MFS	5 (0.96%)	3.80 (0.41, 34.97)	0.239
CRI	16 (3.06%)	7.78 (2.53, 23.92)	0.001
WBC (×109/L)	10.92 ± 3.76	1.00 (0.93, 1.05)	0.980
PLT (×109/L)	195.77 ± 78.14	1.00 (0.97, 1.03)	0.153
Hb (g/L)	125.76 ± 20.60	0.97 (0.95, 0.98)	<0.001
ALT (u/L)	19.95 (13.50-33.38)	1.00 (1.00, 1.00)	0.032
AST (u/L)	19.50 (15.15-28.60)	1.00 (1.00, 1.00)	<0.001
Cr (umol/L)	79.10 (65.45-103.88)	1.00 (1.00, 1.00)	0.005
UA (mmol/L)	317.49 ± 111.70	1.00 (1.00, 1.00)	0.005
TnT (pg/mL)	7.83 (4.50-14.02)	1.00 (1.00, 1.00)	0.002
TG (mmol/L)	1.48 ± 0.85	2.01 (1.41, 2.86)	<0.001
HDL-c (mmol/L)	1.19 ± 0.32	0.46 (0.14, 1.53)	0.202
TG/HDL-c ratio	1.37 ± 0.89	2.04 (1.40, 2.97)	<0.001
Operation			<0.001
No	117 (22.37%)	Ref	
Yes	406 (77.63%)	0.04 (0.02, 0.09)	

Abbreviations: CI: confidence interval; OR: odds ratio; BMI: body mass index; SBP: systolic blood pressure; DBP: diastole blood pressure; WBC: white blood cells count; PLT: platelet count; Hb: hemoglobin; ALT: alanine transaminase; AST: aspartate aminotransferase; Cr: creatinine; UA: uric acid; TnT: troponin T; DM: diabetes mellitus; MFS: Marfan syndrome; CRI: chronic renal insufficiency; TG: triglyceride; HDL-c: high-density lipoprotein cholesterol.

**Table 3:** Relationship between TG/HDL-c ratio and in-hospital mortality in different models.

Exposure	OR (95% CI), *P* value		
	Crude model	Model I	Model II
TG/HDL-c ratio	2.04 (1.40, 2.97), <0.001	2.03 (1.39, 2.95), 0.0002	2.08 (1.32, 3.27), 0.002
TG/HDL-c ratio			
Q1	Ref	Ref	Ref
Q2	1.15 (0.29, 4.58), 0.847	1.17 (0.29, 4.68), 0.824	2.56 (0.55, 11.96), 0.231
Q3	3.77 (1.27, 11.12), 0.017	3.90 (1.31, 11.66), 0.015	4.57 (1.28, 16.37), 0.020
Q4	4.18 (1.43, 12.25), 0.009	3.95 (1.34, 11.61), 0.013	5.29 (1.50, 18.70), 0.010
*P* for trend	<0.0001	<0.0001	<0.0001

Abbreviations: CI: confidence interval; OR: odds ratio; TG/HDL-c: triglyceride/high-density lipoprotein cholesterol. Crude model adjusted for none. Model I adjusted for gender, BMI, and age. Model II adjusted for gender, age, BMI, hypertension, smoking, atherosclerosis, WBC, PLT, Hb, ALT, AST, Cr, UA, and TnT.

**Table 4:** The results of the two-piecewise linear model.

	*N* (%)	OR (95% CI)	*P* value
Fitting model by standard linear regression	523(100%)	2.08 (1.32, 3.27)	0.002
Fitting model by two-piecewise linear regression			
The inflection point of TG/HDL-c ratio			
≤2.05	421 (80.5%)	3.17 (1.54, 6.57)	0.001
>2.05	102 (19.5%)	1.00 (0.80, 1.26)	0.983
*P* for the log-likelihood ratio test	0.017		

Abbreviations: CI: confidence interval; OR: odds ratio. Adjusted for gender, age, BMI, hypertension, smoking, atherosclerosis, WBC, PLT, Hb, ALT, AST, Cr, UA, and TnT.

**Table 5:** Results of subgroup analysis and interaction analysis.

Characteristic	No.	OR (95% CI)	*P* for interaction
Age (years)			0.808
<50	169	1.60 (0.73, 3.53)	
50-60	163	2.01 (1.21, 3.35)	
>60	191	1.63 (1.00, 2.63)	
Gender			0.706
Male	441	1.66 (1.18, 2.33)	
Female	82	2.10 (0.67, 6.57)	
BMI (kg/m^2^)			0.448
<23	214	1.51 (0.32, 7.12)	
23-26.5	216	3.69 (1.01, 13.40)	
>26.5	215	6.67 (0.91, 48.89)	
Smoking			0.039
No	314	2.36 (1.51, 3.68)	
Yes	209	1.18 (0.72, 1.95)	
DM			0.271
No	498	1.82 (1.31, 2.53)	
Yes	25	0.53 (0.04, 7.96)	
Hypertension			0.217
No	137	1.08 (0.44, 2.70)	
Yes	386	1.90 (1.32, 2.74)	
Stroke			0.635
No	507	1.70 (1.22, 2.35)	
Yes	16	1.37 (0.59, 3.19)	
Atherosclerosis			0.749
No	472	1.70 (1.22, 2.36)	
Yes	51	2.20 (0.49, 9.80)	
CRI			0.389
No	507	1.60 (1.13, 2.27)	
Yes	16	1.22 (0.90, 1.65)	
WBC (×10^9^/L)			0.057
<9	173	1.26 (0.75, 2.10)	
9-12	175	3.33 (1.71, 6.50)	
>12	174	1.54 (0.80, 2.98)	
PLT (×10^9^/L)			0.875
<160	174	1.94 (1.16, 3.25)	
160-210	171	1.59 (0.84, 3.00)	
>210	178	1.91 (1.06, 3.45)	
Hb (g/L)			0.406
<120	168	1.57 (1.05, 2.35)	
120-135	175	1.19 (0.46, 3.08)	
>135	180	2.55 (1.19, 5.43)	
ALT (u/L)			0.663
<15	173	2.17 (1.25, 3.78)	
15-25	172	1.43 (0.68, 3.02)	
>25	173	1.77 (1.01, 3.11)	
AST (u/L)			0.571
<15	171	2.15 (1.16, 3.98)	
15-25	172	1.99 (1.12, 3.54)	
>25	176	1.41 (0.78, 2.54)	
Cr (umol/L)			0.801
<70	172	1.99 (0.69, 5.72)	
70-92	174	1.31 (0.62, 2.76)	
>92	174	1.62 (1.06, 2.46)	
UA (mmol/L)			0.618
<5	173	2.29 (1.08, 4.84)	
5-7	172	1.43 (0.80, 2.54)	
>7	173	1.72 (1.05, 2.83)	
Operation			0.020
No	117	2.73 (1.58, 4.71)	
Yes	406	1.04 (0.52, 2.10)	

Abbreviations: CI: confidence interval; OR: odds ratio; BMI: body mass index; WBC: white blood cells count; PLT: platelet count; Hb: hemoglobin; ALT: alanine transaminase; AST: aspartate aminotransferase; Cr: creatinine; UA: uric acid; DM: diabetes mellitus; MFS: Marfan syndrome; CRI: chronic renal insufficiency; TG/HDL-c ratio: triglyceride/high-density lipoprotein cholesterol ratio.

## Data Availability

The datasets used and/or analyzed during the present study were availed by the corresponding author on reasonable request.

## References

[1] Golledge J., Eagle K. A. (2008). Acute aortic dissection. *Lancet*.

[2] Evangelista A., Isselbacher E. M., Bossone E. (2018). Insights from the International Registry of Acute Aortic Dissection. *Circulation*.

[3] Hagan P. G., Nienaber C. A., Isselbacher E. M. (2000). The International Registry of Acute Aortic Dissection (IRAD): new insights into an old disease. *JAMA*.

[4] Trimarchi S., Tolenaar J. L., Tsai T. T. (2012). Influence of clinical presentation on the outcome of acute B aortic dissection: evidences from IRAD. *The Journal of Cardiovascular Surgery*.

[5] Luebke T., Brunkwall J. (2010). Outcome of patients with open and endovascular repair in acute complicated type B aortic dissection: a systematic review and meta-analysis of case series and comparative studies. *The Journal of Cardiovascular Surgery*.

[6] Umaña J. P., Lai D. T., Mitchell R. S. (2002). Is medical therapy still the optimal treatment strategy for patients with acute type B aortic dissections?. *The Journal of Thoracic and Cardiovascular Surgery*.

[7] Golledge J., Kuivaniemi H. (2013). Genetics of abdominal aortic aneurysm. *Current Opinion in Cardiology*.

[8] Wang X., Dong Y., Qi X., Huang C., Hou L. (2013). Cholesterol levels and risk of hemorrhagic stroke: a systematic review and meta-analysis. *Stroke*.

[9] Rist P. M., Buring J. E., Ridker P. M., Kase C. S., Kurth T., Rexrode K. M. (2019). Lipid levels and the risk of hemorrhagic stroke among women. *Neurology*.

[10] Cordero A., Laclaustra M., León M. (2008). Comparison of serum lipid values in subjects with and without the metabolic syndrome. *The American Journal of Cardiology*.

[11] Hokanson J. E., Austin M. A. (1996). Plasma triglyceride level is a risk factor for cardiovascular disease independent of high-density lipoprotein cholesterol level: a meta-analysis of population-based prospective studies. *Journal of Cardiovascular Risk*.

[12] Prasad M., Sara J. D., Widmer R. J., Lennon R., Lerman L. O., Lerman A. (2019). Triglyceride and triglyceride/ HDL (high density lipoprotein) ratio predict major adverse cardiovascular outcomes in women with non-obstructive coronary artery disease. *Journal of the American Heart Association*.

[13] Zhang W., Liu Z., Liu C. (2015). Effect of lipid-modifying therapy on long-term mortality after abdominal aortic aneurysm repair: a systemic review and meta-analysis. *World Journal of Surgery*.

[14] Vallée A., Lelong H., Lopez-Sublet M., Topouchian J., Safar M. E., Blacher J. (2019). Association between different lipid parameters and aortic stiffness. *Journal of Hypertension*.

[15] Powers W. J., Rabinstein A. A., Ackerson T. (2018). 2018 guidelines for the early management of patients with acute ischemic stroke: a guideline for healthcare professionals from the American Heart Association/American Stroke Association. *Stroke*.

[16] Bittner V., Johnson B. D., Zineh I. (2009). The triglyceride/high-density lipoprotein cholesterol ratio predicts all-cause mortality in women with suspected myocardial ischemia: a report from the Women's Ischemia Syndrome Evaluation (WISE). *American Heart Journal*.

[17] Grundy S. M., Cleeman J. I., Daniels S. R. (2005). Diagnosis and management of the metabolic syndrome: an American Heart Association/National Heart, Lung, and Blood Institute scientific statement. *Circulation*.

[18] Ballantyne C. M., Olsson A. G., Cook T. J., Mercuri M. F., Pedersen T. R., Kjekshus J. (2001). Influence of low high-density lipoprotein cholesterol and elevated triglyceride on coronary heart disease events and response to simvastatin therapy in 4S. *Circulation*.

[19] Bhalodkar N. C., Blum S., Enas E. A. (2006). Accuracy of the Ratio of Triglycerides to High-Density Lipoprotein Cholesterol for Predicting Low-Density Lipoprotein Cholesterol Particle Sizes, Phenotype B, and Particle Concentrations Among Asian Indians. *The American Journal of Cardiology*.

[20] Nam K. W., Kwon H. M., Jeong H. Y., Park J. H., Kwon H., Jeong S. M. (2019). High triglyceride/HDL cholesterol ratio is associated with silent brain infarcts in a healthy population. *BMC Neurology*.

[21] Hanak V., Munoz J., Teague J., Stanley A Jr, Bittner V. (2004). Accuracy of the triglyceride to high-density lipoprotein cholesterol ratio for prediction of the low-density lipoprotein phenotype B. *The American Journal of Cardiology*.

[22] Erbel R., Aboyans V., Boileau C. (2015). Corrigendum to: 2014 ESC Guidelines on the diagnosis and treatment of aortic diseases. *European Heart Journal*.

[23] Ahn J. M., Kim H., Kwon O. (2019). Differential clinical features and long-term prognosis of acute aortic syndrome according to disease entity. *European Heart Journal*.

[24] Ma W., Cui C., Feng S. (2019). Serum uric acid and triglycerides in Chinese patients with newly diagnosed moyamoya disease: a cross-sectional study. *BioMed Research International*.

[25] Salazar M. R., Carbajal H. A., Espeche W. G. (2013). Identifying cardiovascular disease risk and outcome: use of the plasma triglyceride/high-density lipoprotein cholesterol concentration ratio versus metabolic syndrome criteria. *Journal of Internal Medicine*.

[26] Barzi F., Patel A., Woodward M. (2005). A comparison of lipid variables as predictors of cardiovascular disease in the Asia Pacific region. *Annals of Epidemiology*.

[27] Chen Z., Chen G., Qin H. (2019). Higher triglyceride to high-density lipoprotein cholesterol ratio increases cardiovascular risk: 10-year prospective study in a cohort of Chinese adults. *Journal of Diabetes Investigation*.

[28] Gaziano J. M., Hennekens C. H., O'Donnell C. J., Breslow J. L., Buring J. E. (1997). Fasting triglycerides, high-density lipoprotein, and risk of myocardial infarction. *Circulation*.

[29] Sonmez A., Yilmaz M. I., Saglam M. (2015). The role of plasma triglyceride/high-density lipoprotein cholesterol ratio to predict cardiovascular outcomes in chronic kidney disease. *Lipids in Health and Disease*.

[30] Deng Q. W., Wang H., Sun C. Z. (2017). Triglyceride to high-density lipoprotein cholesterol ratio predicts worse outcomes after acute ischaemic stroke. *European Journal of Neurology*.

[31] Ma H. B., Wang R., Yu K. Z., Yu C. (2015). Dynamic changes of early-stage aortic lipid deposition in chronic renal failure rats and effects of decorin gene therapy. *Experimental and Therapeutic Medicine*.

[32] Botham K. M., Wheeler-Jones C. P. D. (2013). Postprandial lipoproteins and the molecular regulation of vascular homeostasis. *Progress in Lipid Research*.

[33] Martín-Alonso M., García-Redondo A. B., Guo D. (2015). Deficiency of MMP17/MT4-MMP proteolytic activity predisposes to aortic aneurysm in mice. *Circulation Research*.

[34] Daiber A., Xia N., Steven S. (2019). New therapeutic implications of endothelial nitric oxide synthase (eNOS) function/dysfunction in cardiovascular disease. *International Journal of Molecular Sciences*.

[35] Kaye E. K., Chen N., Cabral H. J., Vokonas P., Garcia R. I. (2016). Metabolic syndrome and periodontal disease progression in men. *Journal of Dental Research*.

